# An Antiapoptotic Neuroprotective Role for Neuroglobin

**DOI:** 10.3390/ijms11062306

**Published:** 2010-05-27

**Authors:** Thomas Brittain, Joanna Skommer, Subadhip Raychaudhuri, Nigel Birch

**Affiliations:** 1 School of Biological Sciences, University of Auckland, 3 Symonds Street, Auckland, NZ, USA; E-Mails: J.Skommer@auckland.ac.nz (J.S.); N.Birch@auckland.ac.nz (N.B.); 2 Department of Biomedical Engineering, 451 Health Sciences Drive, University of California, Davis, CA, USA; E-Mail: raychaudhuri@ucdavis.edu

**Keywords:** neuroglobin, apoptosis, mitochondria, cytochrome *c*, systems biology

## Abstract

Cell death associated with mitochondrial dysfunction is common in acute neurological disorders and in neurodegenerative diseases. Neuronal apoptosis is regulated by multiple proteins, including neuroglobin, a small heme protein of ancient origin. Neuroglobin is found in high concentration in some neurons, and its high expression has been shown to promote survival of neurons *in vitro* and to protect brain from damage by both stroke and Alzheimer’s disease *in vivo*. Early studies suggested this protective role might arise from the protein’s capacity to bind oxygen or react with nitric oxide. Recent data, however, suggests that neither of these functions is likely to be of physiological significance. Other studies have shown that neuroglobin reacts very rapidly with cytochrome *c* released from mitochondria during cell death, thus interfering with the intrinsic pathway of apoptosis. Systems level computational modelling suggests that the physiological role of neuroglobin is to reset the trigger level for the post-mitochondrial execution of apoptosis. An understanding of the mechanism of action of neuroglobin might thus provide a rational basis for the design of new drug targets for inhibiting excessive neuronal cell death.

## 1. Mitochondrial Pathway of Cell Death–A Target for Neuroprotection

**Figure 1:**
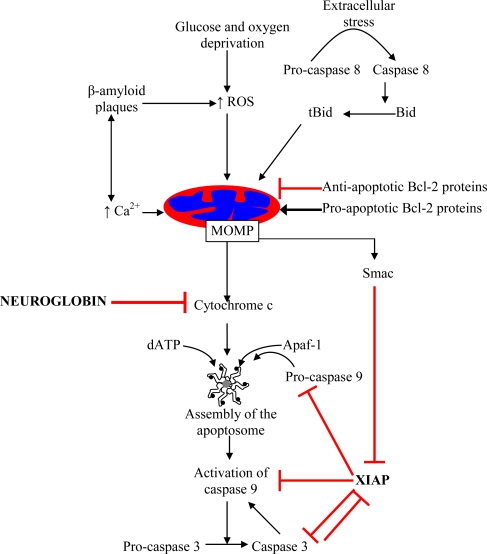
The intrinsic pathway of apoptosis. Mitochondrial pathway of cell death is triggered by a number of internal and external stimuli, and heavily regulated by proteins both up-stream and down-stream of mitochondria. Pre-mitochondrial events that regulate mitochondrial outer membrane permeabilisation (MOMP) in neurons include increase in Ca^2+^ and reactive oxygen species (ROS) levels. MOMP is tightly regulated by the multi-protein Bcl-2 family, which consists of both anti- and pro-apoptotic members. Down-stream of mitochondria, the pathway is regulated by the low-probability event of apoptosome assembly, as well as positive and negative feedback loops involving caspase 3 and 9, and inhibitor of apoptosis proteins such as XIAP. Cytochrome *c* is a key protein, initiating apoptosome assembly and activation of caspase cascade. Neuroglobin binding to and reduction of cytochrome *c* interferes with the mitochondrial pathway of apoptosis immediately down-stream of mitochondria, affecting all types of up-stream stress signals. Lines in red represent inhibitory effects.

A number of neuro-pathological conditions are associated with cell death. In ischemic stroke, vessel occlusion is followed by glucose and oxygen deprivation. In the umbra, immediately surrounding the site of insult, cell death is relatively rapid, whereas in the much larger penumbral region cell death may occur over a period of days. Acute death within the umbra is predominantly by necrosis whilst death within the ischaemic penumbra is apoptotic. The exact fraction of cells which die from apoptosis, as compared with other forms of cell death which are not dependent on caspase activation (e.g., necrosis or cell death dependent on autophagy genes), is variable. Irrespective of the morphological features of neuronal cell death, it is often associated with mitochondrial dysfunctions [1]. Moreover, mitochondrial dysfunctions represent an early event in many neurodegenerative diseases, including Alzheimer’s, Parkinson’s, and Huntington’s disease as well as amyotrophic lateral sclerosis [2]. Multiple proteins regulate the mitochondrial pathway of cell death, both up-stream and down-stream of mitochondria, generating a complex signalling network that functions to buffer limited stress signals and maintain homeostasis, protecting post-mitotic neurons from excessive death. The Bcl-2 family of proteins regulates events up-stream of mitochondria, weighing the pro-survival signals against the stress/damage signals [3]. If the latter prevail, the mitochondrial membrane is permeabilized, which leads to deterioration of the bioenergetic functions of mitochondria, overproduction of reactive oxygen species (ROS), as well as the release of pro-apoptotic molecules such as cytochrome *c* from the inter-membrane space of the mitochondria into the cytosol. Following its release from the mitochondria, cytochrome *c* binds to Apaf-1, and in the presence of ATP promotes oligomerisation of Apaf-1 into the caspase 9-activating platform called the apoptosome (Figure 1). Downstream of mitochondria the pathway is regulated not only by the formation of the apoptosome, but also by positive and negative feedback loops involving active and inactive caspases and a number of additional proteins from the IAP (inhibitor of apoptosis) family, such as X-linked IAP (XIAP) [4] (Figure 1). The cooperative action of all these molecules ensures fail-safe mechanisms, *i.e.*, cells only commit to death if stress signal overcomes all the protective measures, but try to recover if only a portion of pro-survival factors is affected. Identification of new regulatory proteins expressed in neurons may allow for specific intervention in this process, by means of tipping the balance in favour of cell survival, and represents a promising avenue for neuroprotection.

## 2. Neuroglobin Is an Evolutionarily Conserved Neuroprotective Protein

Although the presence of globin protein in the nerves of invertebrates was reported as long ago as 1872 [5] it was not until 2000 that Burmester *et al.* [6] convincingly showed the presence of such a protein in the neural tissues of the mouse, leading to the designation – neuroglobin. Neuroglobin has since been thoroughly characterised and shown to be a 17 kDa molecular weight, single peptide member of the globin super family, exhibiting the same basic structure as the other vertebrate members with a 3 on 3 globin fold, binding a single heme group [7–9]. The main structural difference between neuroglobin and the other members of the family is the presence of a predominantly bis-histidine, 6 co-ordinate, structure at the heme binding site. This form of the protein is in equilibrium with a very small fraction of a mono-histidine, 5 coordinate form which is reactive with all the gaseous ligands normally associated with globin function [10–13].

**Figure 2:**
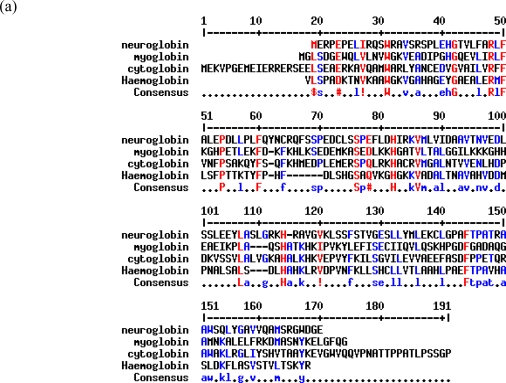

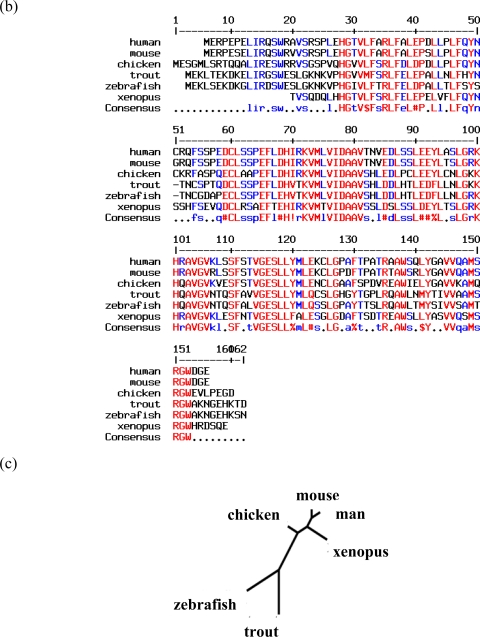
Neuroglobin shares little amino acid sequence homology with other family members and is highly conserved throughout species. Protein alignment of neuroglobin and **(a)** other human globin family members or **(b)** showing homology between species. Residues marked in red match the consensus, residues in blue are weakly conserved. Protein alignments were performed using MultAlin ver 5.4.1 [15]. **(c)** Phylogenetic tree representing conservation of neuroglobin throughout evolution. Phylogenetic analysis was performed using Phylogeny.fr. [16].

Neuroglobin shares little amino acid sequence homology with myoglobin, haemoglobin and cytoglobin (Figure 2a) but its amino acid sequence is highly conserved between species (Figures 2b and c). Phylogenetic analysis indicates a very ancient lineage for this protein, with the other vertebrate globins having diverged from neuroglobin more than 600M years ago [14]. Although neuroglobin has been widely reported as being expressed in brain neurons, its detailed distribution in the mouse brain has only recently been published [17]. From these studies it is clear that neuroglobin is present at high concentration in relatively few areas of the mouse brain, including piriform cortex, amygdale, hypothalamus, arcuate nucleus, habenular nuclei, laterodorsal tegmental nucleus, pedunculopontine tegmental nucleus, locus coeruleus, nucleus of the solitary tract and the spinal trigeminal nucleus, and is expressed at highest concentrations in orexin stimulated neurons [17]. The level of neuroglobin in the human brain has also recently been reported [18]. The highest levels were found in the cerebral cortex and caudatoputamen, and intermediate levels were found in the cerebellum, substantia nigra and medulla, a distribution similar to that previously reported for neuroglobin mRNA [6].

An ancient origin and extreme conservation of neuroglobin’s amino acid sequence, combined with its neuronal localization [6,17], has been taken as indicative of a very specific and crucial role for this protein. Several studies have associated genetic polymorphisms within the human neuroglobin gene with neuroprotection [19–21], and decreased expression of neuroglobin in older people, in women, or associated with single nucleotide polymorphism has been linked to increased risk of Alzheimer’s disease [19]. Together with the population-based genetic association studies, and observations that some forms of neuronal injury are associated with an increased expression of neuroglobin [12], more direct evidence has also been mounting to support the role of neuroglobin in protecting cells from a variety of apoptotic challenges. In cultured cells, ectopic over-expression of neuroglobin has been shown to protect against amyloid beta-hydrogen peroxide-, paraquat-, and HA14-1 (the BH3 mimetic)-induced cell death, as well as from oxygen and glucose deprivation [22–27]. The neuroprotective role of endogenous neuroglobin has been also supported by its knock-down *in vitro*, which renders cortical neuronal cultures more susceptible to hypoxia [19], and decreasing viability of neuroblastoma cells under oxidative stress [28]. Although some questions have been raised concerning the capacity of neuroglobin to provide general protection to neurons *in vivo* [29], other *in vivo* studies support the neuroprotective role of this protein. In neuroglobin-overexpressing transgenic animals the size of cerebral infarct is drastically reduced [30,31]. Similarly, intra-cerebral administration of neuroglobin-expressing adeno-associated virus vector reduced infarct size in rats after focal cerebral ischemia, with opposite effects observed following knock-down of endogenous neuroglobin in rat brains [32]. Contradictions in data derived from *in vivo* experiments almost certainly arises, at least in major part, from differences in the nature, severity, and duration of challenge used in the various studies. Any proposed mechanism of action of neuroglobin, in neurons, thus must consider not only its capacity to provide at least a level of protection to many cell types in the brain but also account for its very nonuniform distribution in the brain.

## 3. Proposed Mechanisms for Neuroglobin-Mediated Neuroprotection

Increased expression of neuroglobin has been shown to protect against stroke and Alzheimer’s disease *in vivo*, and to promote neural survival after oxygen or glucose deprivation, or following oxidative stress. Consequently, a considerable effort has been put into dissecting the molecular mechanisms of neuroglobin’s action under these conditions. Much of the initial work focussed on the myoglobin-like reactivity of the approx 0.1% of the five co-ordinated species present in neuroglobin at equilibrium *in vitro*. These studies proposed that neuroglobin might protect cells by supporting mitochondrial oxidative respiration during time of challenge [33]. Unfortunately, most of these studies relied on the early reports of high, myoglobin like, oxygen affinity of neuroglobin, and ignored the potential for oxygenated neurolgobin to undergo autoxidation [11]. More recent studies, in which autoxidation was suppressed, indicate that neuroglobin’s affinity for oxygen is considerably lower [34]. In fact, under normal physiological conditions, neuroglobin is expected to be only partially saturated with oxygen [34]. These findings, taken together with the relatively low levels of neuroglobin (as compared to myoglobin), even at the sites of its highest expression, have now lead to an abandoning of this hypothesis [35].

Another myoglobin-like reactivity expressed by neuroglobin, namely reaction with nitric oxide (NO), lead to the suggestion that neuroglobin might play a role in NO homeostasis or scavenging [12,36]. The activity of neuronal NO synthases increases after ischemic and traumatic brain injury [37], and the resulting excess NO can rapidly impair mitochondrial functions, leading to mitochondrial permeability, activation of caspase 3 and neuronal cell death [38]. Hence, NO-scavenging activity of neuroglobin could provide neuroprotection. Since this initial suggestion, further studies have identified a rich and complex chemistry for the reactivity of both ferric and ferrous forms of neuroglobin with a range of nitrogen compounds *in vitro.* Apart from reacting with NO, neuroglobin is also involved in nitrite and peroxynitrite chemistry [39] (for which it has unusual reactivity in that it does not react via a ferryl intermediate [40]). However, neuroglobin’s role in detoxification of NO, as envisioned earlier, has been drawn into question by the findings that it is not significantly more reactive than hemoglobin or myoglobin [13]. Furthermore, it has been highlighted that any useful biological reactivity with NO would require the presence of a very effective re-reducing system. Despite many attempts to identify such a system, non has been identified [12,41–43].

Considering the lack of mechanistic explanation of neuroglobin’s neuroprotective effects, further attempts have been recently made to provide a more detailed understanding of its physiological functions. Khan and co-authors have recently suggested that neuroglobin inhibits Pak1 kinase and interacts with RhoGDI and RhoGTPase family members, inhibiting propagation of hypoxia-induced death signal in a form of cytoskeletal reorganisation and mitochondrial aggregation at the sites of raft membrane microdomains [44]. This newly-identified and neuroglobin-regulated death signalling module is formed not only during hypoxia, but also during amyloid beta- and NMDA-induced neuronal cell death [45].

## 4. Reactivity with Cytochrome C

During studies of the redox activities of neuroglobin it was found that very rapid reaction occurs between ferrous neuroglobin and ferric cytochrome *c*. The high rate of intermolecular electron exchange (approx 2,000 s^−1^) is in the range reported for other biologically significant protein-protein redox reactions such as cytochrome c reduction of cytochrome c oxidase [46] or the reduction of hemoglobin or cytochrome c by cytochrome b_5_ [47,48]. This reaction appears to be composed of two steps, namely complex formation followed by electron exchange [49,50]. How might this reaction be of importance in neuroprotection? During the activation of the intrinsic pathway of apoptosis or programmed cell death cytochrome *c* is released into the cytosol, following mitochondrial outer membrane permeabilisation (MOMP) (Figure 1). This process is induced by a plethora of stress signals experienced by neurons, including oxygen or glucose deprivation, increased levels of calcium or ROS, or accumulation of amyloid beta peptide (Figure 1) [1]. Release of cytochrome *c* into the cytosol would then bring neuroglobin and cytochrome *c* into contact. This process would be significantly enhanced by the electrostatic interactions between the two proteins, as cytochrome *c* is an unusually basic protein (pI = 10.2) whilst neuroglobin is an unusually acidic protein (pI = 4.6). Thus, at neurtral pH neuroglobin would be highly negatively charged whilst cytochrome *c* would be very positively charged. The finding that neuroglobin reacts very rapidly with cytochrome *c* lead to the hypothesis that neuroprotection by neuroglobin arises from its intervention in the intrinsic pathway of apoptosis following the release of cytochrome *c* into the cytosol [51].

## 5. Experimental Evidence for Cytochrome c-Neuroglobin Interaction

**Figure 3:**
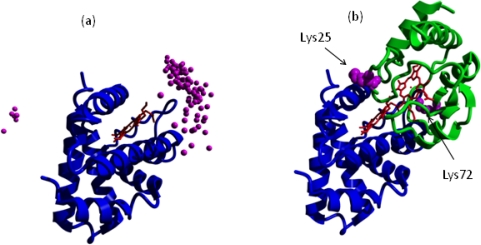
Cytochrome c binding to neuroglobin. **(a)** Shows the ensemble of complexes calculated from soft docking calculations. The centres of mass of the docked cytochrome c molecules are indicated by the small spheres. **(b)** Shows the structure of the lowest energy docked complex with neuroglobin in blue and cytochrome c in green.

In the absence of direct structural determinations of the complex formed between cytochrome *c* and neuroglobin, computational methods have been used to identify putative structures for this complex. Application of soft docking algorithms to this problem has predicted the production of an ensemble of closely related structures, which share a common binding site but exhibit a small degree of slippage across this binding interface [50] (Figure 3). When other potential heme binding partners, such as myoglobin, were studied in the same system no such specific docking was observed. Close examination of the lowest energy complex has allowed the identification of amino acids likely to be involved in the docking process. In particular Lys25 and Lys72 appear to play a significant role in the interface between neuroglobin and cytochrome *c* (Figure 3). It is interesting to note that these two amino acids have previously been identified as key residues in the interaction of cytochrome *c* with Apaf-1 [52–55] – the major cytosolic protein involved in apoptosome assembly. Experimental evidence for the formation of a cytochrome *c*-neuroglobin complex in solution has been obtained using both NMR and surface plasmon resonance studies [50]. These experiments indicate the formation of a weakly bound complex (K ∼ 100μM) which is sensitive to both temperature and ionic strength.

The ability of neuroglobin to interact with cytochrome *c* and hence interfere with the process of apoptosis, via inhibition of apoptosome assembly, has also been studied more directly. Using cell lysates devoid of mitochondria but containing Apaf-1, it has been demonstrated that although neuroglobin itself has no capacity to affect apoptosome assembly, in its presence the pro-apoptotic activity of cytochrome *c* is lost [26]. These biochemical findings have been further supported by cellular studies in human neuroblastoma SH-SY5Y cells treated with the BH3 mimetic (Bcl-2 inhibitor), HA14-1. In response to HA14-1, SH-SY5Y cells undergo the mitochondrial pathway of apoptosis, associated with the loss of mitochondrial transmembrane potential and activation of initiator caspase 9. This specifically-induced mitochondrial pathway of apoptosis is significantly blocked by ectopically-overexpressed neuroglobin, with the extent to which neuroglobin increases survival of HA14-1-treated cells depending on the strength of stress. While micromolar levels of neuroglobin are capable of nearly completely blocking cell death induced by a low-level BH3 mimetic-induced stress signal, rapid apoptosis triggered by high-level stress is blocked only partially. Considering the complexity of the apoptotic network, analysis of the role of neuroglobin in regulation of apoptosis, depending on the concentration of this protein as well as varying stress, is not easily accessible experimentally.

Recent developments in the field of computational systems level biology of apoptotic pathways open new doors to the modeling of cellular responses and their dependence on protein concentration and type/strength of stress. Using Monte Carlo computational modeling of apoptotic signaling, based on measured reaction rate constants, where diffusion and reaction of signaling molecules are simulated at an individual molecular level, it is possible to explicitly simulate molecular interactions and model spatial heterogeneity such as the localization of pro- and anti-apoptotic proteins on mitochondrial membranes or formation of multi-protein apoptosome complexes [56]. Models based on ordinary differential equations (ODEs) which have previously been employed [57] cannot simulate such important details of apoptotic signaling. More importantly, ODE-based models cannot capture cell-to-cell stochastic variability in apoptotic signaling that arise solely due to the inherent stochastic nature of chemical reactions. Careful validation of the key findings of such Monte Carlo computational models, such as the time scale of apoptosis, all-or-none caspase activation with large cell-to-cell variability, and the post-mitochondrial origin of cell-to-cell variability in the execution of the mitochondrial pathway of apoptosis, has allowed the application of such models to the study of the impact of neuroglobin on apoptotic networks in more detail [26,56].

These studies indicate that, thanks to its extremely rapid redox reaction with cytochrome c, neuroglobin can inhibit activation of caspases, associated with the inhibition of the low probability event of apoptosome formation, and that the activation of executioner caspase 3 is very sensitive to the ratio of neuroglobin and cytochrome *c* concentrations [26]. As the concentration of cytosolic cytochrome *c* increases, which mimics a situation observed in highly stressed cells, an increasingly higher concentration of neuroglobin is needed to block activation of caspases. The required protective concentration of neuroglobin depends on the increases in cytochrome *c* concentrations in a non-linear manner [26]. Using the same computational approach it has been shown that a higher level of cytosolic neuroglobin is needed to completely abolish apoptotic activation in the absence of redox reaction between cytochrome *c* and neuroglobin [26]. In the future, extended model calculations will help to elucidate the co-operation between neuroglobin and other anti-apoptotic proteins that act down-stream of mitochondria, for example the inhibitor of apoptosis (IAP) proteins.

## 6. Hypothesis: A Co-Ordinating Role for Neuroglobin

**Figure 4:**
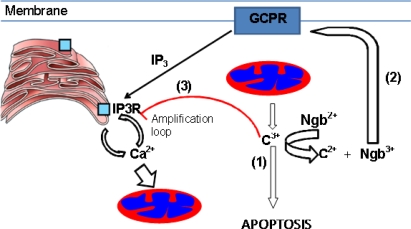
A co-ordinating role for neuroglobin. The diagram shows the three actions of neuroglobin: (1) in suppressing apoptosis by binding and reducing cytochrome *c* released from mitochondria, (2) in inhibiting the production of IP3 by GPCR and (3) maintaining the auto-feedback loop, which limits the release of calcium from endoplasmic reticulum (ER) via type 2 IP3 receptors by binding cytochrome *c*.

The reaction of neuroglobin with cytochrome *c* might well have multiple effects on apoptosis (Figure 4). In terms of direct action on apoptotic signaling, the reaction of ferrous neuroglobin with ferric cytochrome *c*, released from the mitochondria, would produce ferrous cytochrome *c*. This reaction would thus convert pro-apoptotic ferric cytochrome *c* into non-apoptotic ferrous cytochrome *c* [58–60], stopping apoptosome formation and hence halting apoptosis. During the normal process of activation of the intrinsic pathway of apoptosis cytochrome *c* released from mitochondria suppresses the auto-inhibition of calcium release from type 1 IP3 receptors in the ER [61]. This initiates a feed forward amplification cycle, as raised calcium levels induce other mitochondria to release further amounts of cytochrome *c*, and so an apoptotic avalanche is created. In the presence of neuroglobin the initially released cytochrome *c* is sequestered by the neuroglobin and the apoptotic avalanche avoided. *In vivo* support for such a function of neuroglobin in suppression of cytosolic calcium levels during apoptosis can be found in the work of Duong *et al.* [62] and Liu *et al.* [63]. These authors showed that apoptotic challenge of SH-SY5Y cells and cultured transgenic mouse cortical neurons over-expressing neuroglobin lead to suppression of cytosolic clalcium levels whilst maintaining mitochondrial membrane potential and cytosolic ATP concentrations. Further more on reaction with ferric cytochrome *c* ferrous neuroglobin is converted to ferric neuroglobin. Ferric neuroglobin has been identified as a potent inhibitor of GPCR and hence IP3 production [64]. The reaction of ferrous neuroglobin with ferric cytochrome *c* thus produces another factor responsible for the suppression of increased cytosolic calcium concentration and suppresses apoptosis. The levels of neuroglobin indicated as protective by computational studies are consistent with those found in nerve cells, and may have the capacity to protect the cells from mild challenge such as those arising from calcium fluctuations encountered during normal nerve function. In support of this hypothesis, neuroglobin is found at high concentrations in neurons stimulated with orexin, an excitatory neuropeptide which increases intracellular Ca^2+^ concentration in neurons together with amyloid peptide, both pro-apoptotic stimuli [65].

## 7. Neuroglobin in Brain Tumours: Dr Jekyll or Mr Hyde?

The strategy of blocking apoptosis operates also in cancer cells, which by virtue of over-expression of anti-apoptotic proteins survive despite being subjected to harsh environmental conditions or intracellular pro-apoptotic signalling [66]. If neuroglobin-mediated protection from cell death is of physiological relevance, one could expect that cancer cells will also hijack this mechanism to advance their survival. In agreement with this hypothesis, a recent particular study has shown that neuroglobin is up-regulated in hypoxic microregions of glioblastoma tumour xenografts [67].

Brain tumours are usually resistant to conventional chemotherapeutics that trigger apoptosis up-stream of mitochondria, but remain sensitive to post-mitochondrial induction of apoptosis [68]. This indicates that despite over-expression of anti-apoptotic proteins that regulate MOMP, apoptotic signalling down-stream of mitochondria remains functional in these cancer cells. An increased expression of Apaf-1 has been detected in high-grade astrocytomas, medulloblastomas, and gliomas as compared to adjacent normal neural tissue [68]. This leads to hyperactivation of post-mitochondrial events of the intrinsic pathway of apoptosis, as illustrated by the fact that glioblastoma and medulloblastoma cell lines are more sensitive to the impact of cytosolic cytochrome *c* than are normal neural cells [68]. It can be thus hypothesized that not only increased Apaf-1 expression, but also decreased expression of neuroglobin, could contribute to the high sensitivity of brain tumours to post-mitochondrial induction of apoptosis. This has not yet been investigated, but analysis of gene profiling data from Sun *et al*. [69]*,* accessed through the Oncomine Gene Profiling Database, demonstrates a statistically significant decrease in neuroglobin mRNA levels in glioblastoma, oligonendroglioma and astrocytoma as compared with brain from epilepsy patients. Undoubtedly further studies are required to dissect the role of neuroglobin in tumorigenesis and sensitivity to chemotherapy, and to identify potential of interfering in neuroglobin-cytochrome *c* interaction in cancer cells for therapeutic purposes.

## 8. Surviving the Release of Cytochrome *c*: Neuroglobin as a Drug Target

Delayed or aborted apoptosis can allow survival of cells under stress. Whether blocking apoptosis can rescue neurons and allow their recovery from the loss of mitochondrial transmembrane potential may well depend on the type and strength of stress signal. Several lines of evidence suggest, however, that survival of neurons can be significantly enhanced providing formation of apoptosome/activation of caspases is blocked [70]. Therefore, blocking immediate post-mitochondrial events, such as cytochrome *c*-mediated formation of the apoptosome, could be a widely applicable target for neuroprotection. In the future efforts will be directed towards understanding the structural requirements for neuroglobin-cytochrome *c* interactions, and the design of neuroglobin mimetics that will render cytochrome *c* apoptotically inactive.

## 9. Conclusions

Neuroglobin is an evolutionarily highly conserved protein localized in brain neurons where it protects from a variety of insults, such as oxidative stress or amyloid beta accumulation. The neuroprotective role of neuroglobin has been shown both *in vitro* and *in vivo*, and its decreased expression has been associated with increased risk of Alzheimer’s disease. The quest for understanding how neuroglobin interferes with neuronal cell death has led to several hypotheses. Recent studies indicate that neuroglobin can interfere with propagation of cell death signaling mediated by RhoGTPases, as well as bind to ferric cytochrome *c* and inhibit its apoptotic activity. Neuroglobin emerges as a critical player that regulates immediate post-mitochondrial events in the intrinsic pathway of apoptosis, opening new avenues for therapeutic interventions in numerous neurological disorders.
